# Supplementary material for: Transcriptomic Insights into Mechanisms of Early Seed Maturation in the Garden Pea (Pisum sativum L.)
Source: Cells. 2020 Mar 23;9(3):779. doi: 10.3390/cells9030779 (PMC7140803; doi:10.3390/cells9030779)

Sprint−2(20) vs Zhongv

Global Summary

%DE = 0.79

genes with fdr < 0.2 = 458 (231 + / 227 −)

genes with fdr < 0.1 = 377 (183 + / 194 −)

genes with fdr < 0.05 = 343 (164 + / 179 −)

genes with fdr < 0.01 = 182 (59 + / 123 −)

genes in genesets = 0

<FC> = 0

<t−score> = −1.69

<p−value> = 0.01

<fdr> = 0.21

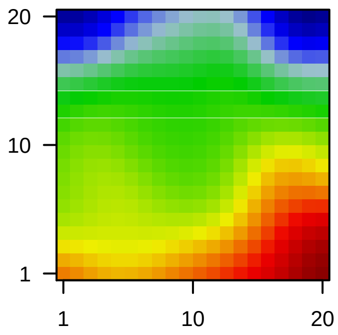

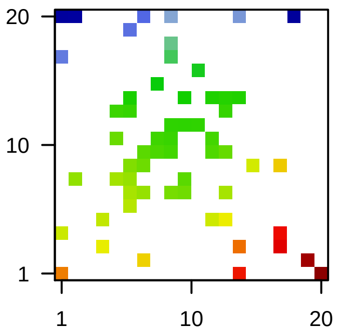

Global Genelist

Rank	ID	log(FC)	p−value / fdr	Metagene	Description
1	TRINITY_DN1	−0.03	0.0000000200001	10 x 7	
2	TRINITY_DN2	0	0.0000000290001	9 x 10	
3	TRINITY_DN5	−0.2	0.0000000530001	13 x 5	
4	TRINITY_DN5	−0.2	0.0000000560001	9 x 20	
5	TRINITY_DN4	0	0.0000000580001	9 x 11	
6	comp48352_c	−0.05	0.0000000580001	10 x 14	
7	TRINITY_DN6	0.24	0.0000000750001	13 x 14	
8	TRINITY_DN4	−0.03	0.0000000780001	12 x 10	
9	TRINITY_DN1	0	0.0000000790001	9 x 10	
10	TRINITY_DN4	0	0.0000000860001	10 x 7	
11	TRINITY_DN3	0	0.0000001090001	9 x 11	
12	TRINITY_DN2	0	0.0000001120001	8 x 10	
13	TRINITY_DN1	0	0.0000001160001	9 x 11	
14	TRINITY_DN5	0	0.0000001280001	9 x 11	
15	TRINITY_DN4	−0.15	0.0000001370001	7 x 2	
16	TRINITY_DN1	0	0.0000001500001	9 x 11	
17	comp39525_c	0.27	0.0000001550001	6 x 8	
18	TRINITY_DN7	0	0.0000001620001	9 x 11	
19	TRINITY_DN3	0.45	0.0000001670001	19 x 2	
20	TRINITY_DN1	0	0.0000001720001	9 x 11	

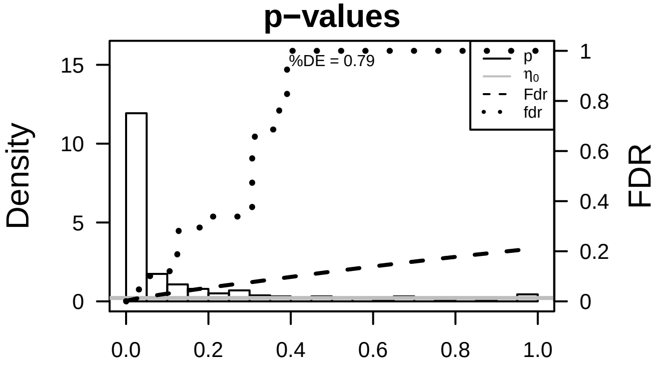

Supplement: Supplementary file 1 [file cells-09-00779-s001.zip › Supplementary materials/FileS3.zip!/FileS2/Hormones - Results/Summary Sheets - Differences/Reports/Sprint.2.20..vs.Zhongwan.6.25..pdf]
